# Dynamics of a network mediated by IL-36 and involved in the pathogenesis of psoriasis

**DOI:** 10.3389/fnetp.2024.1363791

**Published:** 2024-05-31

**Authors:** Sneha Pandey, Syona Tiwari, Sulagna Basu, Rajiv Kumar Mishra, Rakesh Pandey

**Affiliations:** ^1^ Bioinformatics, MMV, Banaras Hindu University, Varanasi, India; ^2^ iOligos Technologies Private Limited, Noida, India

**Keywords:** skin diseases, keratinocytes, dendritic cells, population dynamics, autoimmune diseases, bistability, systems biology, mathematical biology

## Abstract

The pathogenesis of the inflammatory, chronic, and common skin disease psoriasis involves immune cells, skin cells (keratinocytes), and the cytokines they secrete. Hyperproliferation and abnormal differentiation of keratinocytes are hallmarks of the disease. The roles of cytokines such as TNF*α*, IL-15, IL-17, and IL-23 in psoriasis have been studied through mathematical/computational models as well as experiments. However, the role of proinflammatory cytokine IL-36 in the onset and progression of psoriasis is still elusive. To explore the role of IL-36, we construct a network embodying indirect cell–cell interactions of a few immune and skin cells mediated by IL-36 based on existing knowledge. We also develop a mathematical model for the network and perform a global sensitivity analysis. Our results suggest that the model is most sensitive to a parameter that represents the level of cytokine IL-36. In addition, a steady-state analysis of the model suggests that an increase in the level of IL-36 could lead to the hyperproliferation of keratinocytes and, thus, psoriasis. Our analysis also highlights that the plaque formation and progression of psoriasis could occur through either a gradual or a switch-like increase in the keratinocyte population. We propose that the switch-like increase would be due to a bistable behavior of the network toward either a psoriatic or healthy state and could be used as a novel treatment strategy.

## 1 Introduction

Psoriasis is one of the most common skin diseases, and approximately 2%–3% of the world’s population is affected by it (Parisi et al., 2013). It is inflammatory and chronic in nature, distinguished by the hyperproliferation of keratinocytes (skin cells) and infiltration of immune cells in the psoriatic lesions. The lesion could be scaly or in the form of pustules, characterizing different types of psoriasis. The involvement of immune cells, such as T-lymphocytes, dendritic cells, macrophages, mast cells, and neutrophils, in the pathophysiology of psoriasis has been well-established (Lowes et al., 2014). The interaction among these immune and skin cells is believed to be mediated by cytokines. Recently, the roles of cytokines TNF*α*, IL-15, and the IL-17/IL-23 axis have been demonstrated by constructing networks of indirect cell–cell interactions among immune cells and keratinocytes (Oza et al., 2017; Pandey et al., 2023). Based on the steady-state behavior of the mathematical models of these networks, authors have demonstrated how an increase in the level of modeled cytokines could lead to the progression of psoriasis. Cytokines involved in psoriasis vulgaris have been more extensively explored than those in other types of psoriasis (Chen et al., 2021; Sachen et al., 2022), such as generalized pustular psoriasis (GPP) in which inflammatory cytokine IL-36 is believed to have a key role (Johnston et al., 2017). However, the underlying mechanism of its role in the pathogenesis of psoriasis vulgaris is still elusive. To address that, we have constructed a network for the indirect cell–cell interactions among immune and keratinocyte cells mediated by the cytokine IL-36. Our network is built on the published observations about the interactions of epidermal keratinocytes, T cells, macrophages, and dendritic cells (DC). Modulation of cells through IL-36 occurs upon binding of IL-36 to the receptors present on the surface of modeled cells in the network. Keratinocytes (K) are a predominant source of IL-36 cytokines, and in an autocrine manner, they enhance the expression of IL-36 (Carrier et al., 2011). A skin injury in the presence of psoriasis stimulates the release of IL-36 by neighboring keratinocytes in a paracrine manner by releasing cathelicidin LL-37 from dead keratinocytes (Li et al., 2014; Zhang et al., 2016). Due to that, T cells and dendritic cells are recruited at the injury site (Samotij et al., 2021). The receptors of IL-36 are predominantly present on naive *CD*4^+^ T cells, and one of the isoforms of the cytokine, IL36*β*, is also constitutively expressed in these cells (Vigne et al., 2011). Many studies have reported that the *CD*4^+^ T cells present in the psoriatic lesion hyperproliferate in response to an IL-36 stimulus (Günther and Sundberg, 2014). It has been observed that bone marrow-derived dendritic cells show Th1 responses as a result of IL-36 induction (Vigne et al., 2011). The DCs activated by IL-36 secrete IL-12, and a synergistic effect of both cytokines results in the polarization of naive *CD*4^+^ *T* cells toward Th1 (Vigne et al., 2011). In addition, IL-36 promotes Th17 and Th22 lymphocyte polarization through a similar DC-mediated mechanism (Mercurio et al., 2018; Samotij et al., 2021). It is also observed that IL-36-mediated T-cell activation by dendritic cells leads to the hyperproliferation of keratinocytes (Foster et al., 2014).

Macrophages are also one of the sources of IL-36 in a psoriatic lesion (Dietrich et al., 2016). Macrophages treated with IL36*γ* stimulated the release of TNF*α* and IL-23. These cytokines have multiple roles, such as stimulating the differentiation and proliferation of T cells (Bridgewood et al., 2018), inducing cytokine release by keratinocytes (as illustrated by (D’erme et al., 2015)), and promoting the maturation of dendritic cells (Sachen et al., 2022). Human dermal macrophages have a high concentration of IL-36R on their surface, which is converted into a proinflammatory form (M1 phenotype) upon the action of IL-36 (Dietrich et al., 2016). Cytokine-stimulated macrophages increase the adherence of monocytes to endothelial cells and cause the polarization of lymphocytes, in this case, T cells, due to the upregulation of the IL-17/IL-23 axis (Bridgewood et al., 2018).

**FIGURE 1:**
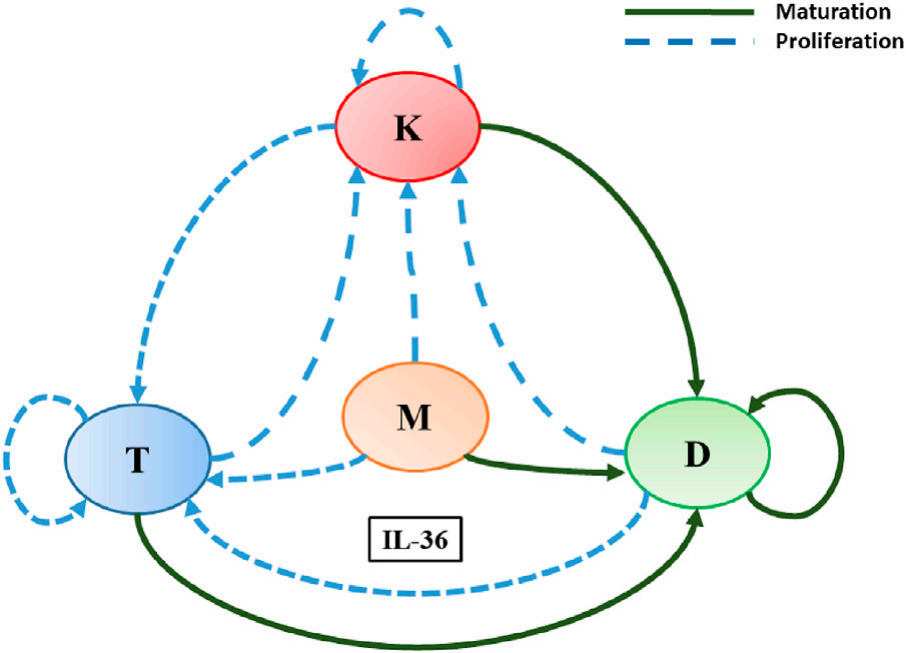
A schematic diagram explaining IL-36-mediated interactions among immune cells and keratinocytes involved in the pathogenesis of psoriasis. Here, L, K, D, and M denote T-lymphocytes, keratinocytes, dendritic cells, and macrophages, respectively. Different colored arrows represent different types of interactions among the cells involved.

These indirect cell–cell interactions mediated by IL-36 led to the construction of a network, as illustrated in Figure 1. Sources and effect of IL-36 cytokines on the modelled cells presented in the network are summarized in Table 1. Here, we have developed a mathematical model for the constructed network mediated by IL-36 to explore the steady-state behavior of the network. We have denoted the IL-36 agonists (*α*, *β*, and *γ*) through a single IL-36 cytokine as they bind to the same signaling receptor (IL-36R) and are expressed in all the cell types in the model. The three separate cytokine agonists lead to the pathogenesis of psoriasis through hyperproliferation and, in some cases, maturation of the modeled immune cells. We have denoted them by a single cytokine that binds to the cell surface receptors IL-36R on the modeled immune cells to keep the model simple and remain trackable. We conducted a global sensitivity analysis to explore the impact of changing model parameters and to assess the sensitivity of the model outcome to each parameter. Our results demonstrate that an increase in IL-36 could lead to the hyperproliferation of keratinocytes, which is considered a hallmark of psoriasis. We also observed that the route of the plaque formation and or progression of psoriasis could be via a gradual or switch-like increase in the population of keratinocytes. This switch-like behavior could be used as a new treatment strategy, as argued in a few recent studies (Pandey et al., 2021; Pandey et al., 2023).

**TABLE 1:** IL-36 cytokines with their source and effect on different cell types. Here, **S** represents the origin of the cytokines, while **+** denotes a net increase in the cell population. These changes are outcomes of the indirect cell–cell interactions via modeled cytokines, that is, IL-36 agonists. The agonists represent the three receptor agonists that are members of the IL-36 cytokine family, that is, IL-36α, IL-36β, and IL-36γ. They initiate the IL-36 receptor signaling and activate various proinflammatory mediators like **NF**κ**B** and MAPK.

Cytokine	Keratinocytes	T-Cell	Dendritic cell	Macrophages
IL-36 Cytokine **(agonists: IL-36** *α* **,** *β* **, and** *γ* **)**	**+** (Foster et al., 2014), **S** (Carrier et al., 2011; Li et al., 2014; D’erme et al., 2015)	**+** (Vigne et al., 2011; Bridgewood et al., 2018; Mercurio et al., 2018; Samotij et al., 2021)	**+** (Samotij et al., 2021)	**S** (Dietrich et al., 2016)

## 2 Materials and methods

Based on the available published knowledge about the role of cytokine IL-36 in the pathogenesis of psoriasis and GPP, a schematic diagram is prepared to depict the indirect cell–cell interactions among skin cells (keratinocytes) and immune cells (dendritic, T cells, and macrophages) (Figure 1). Interactions depicted in the diagram elucidate the involvement of IL-36 in the proliferation, maturation, and differentiation of modeled cells in a psoriatic lesion. In a recent study, Pandey et al. (2021) developed a mathematical model and demonstrated the involvement of cytokines TNF*α*, IL-23/IL-17, and IL-15 in the pathogenesis of psoriasis (Pandey et al., 2021). The mathematical modeling framework they proposed is ideal for the present study. Therefore, we follow that framework for modeling the network mediated by IL-36 as follows.

### 2.1 Mathematical modeling framework

In our model, we consider the lesional skin of a psoriatic patient with a resident population of keratinocytes and immune cells (T cells, dendritic cells, and macrophages). We assume that all the modeled cells can be infiltrated into this lesion. This region of skin will be treated with different levels of IL-36 to investigate the role of that cytokine in the plaque formation and progression of psoriasis.

The dynamics of the modeled cell population are governed by following ordinary differential equations (ODEs) if the framework proposed elsewhere (Pandey et al., 2021) is applied:

$$\frac{dx}{dt}=\alpha_{x}+\beta \left(x\right)-\gamma_{x}x$$ (1)

Here, *x* represents the population of a modeled cell type that is, *x* = {*K*, *D*, *T*, *M*} where the populations of keratinocytes, dendritic cells, T cells, and macrophages are denoted by K, D, T, and M, respectively. The parameter *α*_*x*_ denotes a fixed rate of increase in the population of modeled cells *x*) through their migration toward the lesion or their differentiation/maturation in the lesion (independent of the cytokine IL-36). *β* denotes the net rate of change in the cell population that accounts for the cumulative effects of the cytokine in cell differentiation, maturation, and proliferation. The rate of decrease in the cell population through other processes, including apoptosis not mediated by IL-6, is denoted by the parameter *γ*_*x*_. The function *β* is considered to be a function that saturates at *β*_*x*_ and depends on the population of all modeled cells or a few based on the interaction network shown in Figure 1. This dependence is modeled through the following two forms of *β*.

(i) when *β* increases due to the modulatory effect of the population of all cell types or a few

$$\beta =\beta_{x}\frac{x^{n}}{k_{x}^{n}+x^{n}}$$ (2)

(ii) when *β* decreases because of the modulatory effect of the population of all cell types or a few

$$\beta =\beta_{x}\frac{k_{x}^{n}}{k_{x}^{n}+x^{n}}.$$ (3)

In Eqs 2, 3, *β*_*x*_ represents the saturated value of function *β* due to the modulatory effect of modeled cells. Here, the parameter *k*_*x*_ represents the population of a modeled cell type that is required to show the half-maximal change of it or another cell’s population, depending on the interaction of the network shown in Figure 1. The slope of the function *β* is determined by the Hill-coefficient like parameter *n*. The rationale behind using the Hill-like function here is its wide acceptance as an empirical saturating function.

### 2.2 Indirect cell–cell interaction network mediated by cytokine IL-36

All modeled indirect cell–cell interactions driven by IL-36 have been depicted in Figure 1 in the form of a schematic network. The following system of ordinary differential equations will be obtained if the above-mentioned modeling framework is applied to model this interaction network.

$$\begin{array}{cc} \frac{dD}{dt} & =\alpha_{D}+\beta_{IL36}\left(\frac{D^{n}}{k_{D}^{n}+D^{n}}\frac{T^{n}}{k_{T}^{n}+T^{n}}\frac{M^{n}}{k_{M}^{n}+M^{n}}\frac{K^{n}}{k_{K}^{n}+K^{n}}\right)-\gamma_{D}D\\ \frac{dK}{dt} & =\alpha_{K}+\beta_{IL36}\left(\frac{D^{n}}{k_{D}^{n}+D^{n}}\frac{T^{n}}{k_{T}^{n}+T^{n}}\frac{M^{n}}{k_{M}^{n}+M^{n}}\frac{K^{n}}{k_{K}^{n}+K^{n}}\right)-\gamma_{K}K\\ \frac{dT}{dt} & =\alpha_{T}+\beta_{IL36}\left(\frac{D^{n}}{k_{D}^{n}+D^{n}}\frac{T^{n}}{k_{T}^{n}+T^{n}}\frac{M^{n}}{k_{M}^{n}+M^{n}}\frac{K^{n}}{k_{K}^{n}+K^{n}}\right)-\gamma_{T}T\\ \frac{dM}{dt} & =\alpha_{M}-\gamma_{M}M \end{array},$$ (4)

where D, K, T, and M denote the population of dendritic cells, keratinocytes, T cells, and macrophages, respectively. The parameter *β*_*IL*36_ represents the level of cytokine IL-36. The parameter *k*_*D*_ represents the population of dendritic cells in the lesion required to attain the half-maximal effect of dendritic cells (D) on D, K, and T (illustrated in the network 1). Similarly, *k*_*K*_, *k*_*T*_, and *k*_*M*_ denote the population of keratinocytes, T cells, and macrophages, respectively, which are required for the half-maximal effect of the K, T, and M populations on modeled cells. The parameters *α*_*D*_, *α*_*K*_, *α*_*T*_, and *α*_*M*_ represent the rates of migration (in cells/day) of dendritic cells, keratinocytes, T cells, and macrophages, respectively, to the site of psoriatic lesion. Their rate of apoptosis (in *day* ^−1^) is represented by *γ*_*D*_, *γ*_*K*_, *γ*_*T*_, and *γ*_*M*_, respectively. The aforementioned set of ODEs indicates that the dynamics of the macrophage population are independent of the population of other cell types (D, K, and T). Hence, a quasi-steady state is assumed for macrophages, and the dynamics of the system of ordinary differential equations are governed by the following system of equations.

$$\begin{array}{cc} \frac{dD}{dt} & =\alpha_{D}+\beta_{IL36}\left(\frac{D^{n}}{k_{D}^{n}+D^{n}}\frac{T^{n}}{k_{T}^{n}+T^{n}}\frac{M^{*n}}{k_{M}^{n}+M^{*n}}\frac{K^{n}}{k_{K}^{n}+K^{n}}\right)-\gamma_{D}D\\ \frac{dK}{dt} & =\alpha_{K}+\beta_{IL36}\left(\frac{D^{n}}{k_{D}^{n}+D^{n}}\frac{T^{n}}{k_{T}^{n}+T^{n}}\frac{M^{*n}}{k_{M}^{n}+M^{*n}}\frac{K^{n}}{k_{K}^{n}+K^{n}}\right)-\gamma_{K}K\\ \frac{dT}{dt} & =\alpha_{T}+\beta_{IL36}\left(\frac{D^{n}}{k_{D}^{n}+D^{n}}\frac{T^{n}}{k_{T}^{n}+T^{n}}\frac{M^{*n}}{k_{M}^{n}+M^{*n}}\frac{K^{n}}{k_{K}^{n}+K^{n}}\right)-\gamma_{T}T \end{array}$$ (5)

Here, $M^{*}=\frac{\alpha_{M}}{\gamma_{M}}$ denotes the macrophage population at a steady state. To further reduce the complexity of the system of ODEs, we make the system dimensionless by assuming $\bar{K}=\frac{K}{k_{K}}$ , $\bar{T}=\frac{T}{k_{T}}$ , $\bar{D}=\frac{D}{k_{D}}$ , and $\bar{M}=\frac{M^{*}}{k_{M}}$ . The dimensionless ODE system reads as

$$\begin{array}{cc} \frac{d\bar{D}}{dt} & ={\alpha \prime }_{D}+{\beta \prime }_{IL36}\left(\frac{{\bar{D}}^{n}}{1+{\bar{D}}^{n}}\frac{{\bar{T}}^{n}}{1+{\bar{T}}^{n}}\frac{{\bar{M}}^{n}}{1+{\bar{M}}^{n}}\frac{{\bar{K}}^{n}}{1+{\bar{K}}^{n}}\right)-\gamma_{D}\bar{D}\\ \frac{d\bar{K}}{dt} & ={\alpha \prime }_{K}+{\beta \prime }_{IL36}\cdot e\cdot \left(\frac{{\bar{D}}^{n}}{1+{\bar{D}}^{n}}\frac{{\bar{T}}^{n}}{1+{\bar{T}}^{n}}\frac{{\bar{M}}^{n}}{1+{\bar{M}}^{n}}\frac{{\bar{K}}^{n}}{1+{\bar{K}}^{n}}\right)-\gamma_{K}\bar{K}\\ \frac{d\bar{T}}{dt} & ={\alpha \prime }_{T}+{\beta \prime }_{IL36}\cdot f\cdot \left(\frac{{\bar{D}}^{n}}{1+{\bar{D}}^{n}}\frac{{\bar{T}}^{n}}{1+{\bar{T}}^{n}}\frac{{\bar{M}}^{n}}{1+{\bar{M}}^{n}}\frac{{\bar{K}}^{n}}{1+{\bar{K}}^{n}}\right)-\gamma_{T}\bar{T} \end{array}$$ (6)

where ${\alpha \prime }_{D}=\frac{\alpha_{D}}{k_{D}}$ , ${\beta \prime }_{IL36}=\frac{\beta_{IL36}}{k_{D}}$ , ${\alpha \prime }_{K}=\frac{\alpha_{K}}{k_{K}}$ , $e=\frac{k_{D}}{k_{K}}$ , ${\alpha \prime }_{T}=\frac{\alpha_{T}}{k_{T}}$, and $f=\frac{k_{D}}{k_{T}}$ .

### 2.3 Model parameters

The typical values of all the model parameters used for obtaining numerical solutions of the system of ODEs are summarized in Table 2. The values for the rate of apoptosis of T cells (*γ*_*T*_), dendritic cells (*γ*_*D*_), and keratinocytes (*γ*_*K*_) are taken from a recent study (Pandey et al., 2021). The values of the remaining parameters were assumed subsequent to a systematic model exploration and calibration. Because the dependence of model results is explored through bifurcation analysis, the exact value of each parameter is not required.

**TABLE 2:** Default values of the model parameters.

Parameters	Values	Biological meaning
${\alpha \prime }_{K}$	0.1 day^−1^	Effective rate of migration of keratinocytes toward the lesion
${\alpha \prime }_{D}$	1 day^−1^	Effective rate of migration of dendritic cells toward the lesion
${\alpha \prime }_{T}$	0.5 days^−1^	Effective rate of migration of T cells toward the lesion
$\beta_{IL36}^{\prime }$	4 days^−1^	Denotes the level of IL-36 cytokine
*γ*_*K*_	0.5 days^−1^	Rate of apoptosis of keratinocytes
*γ*_*D*_	0.2 days^−1^	Rate of apoptosis of dendritic cells
*γ*_*T*_	0.5 days^−1^	Rate of apoptosis of T cells
$\bar{M}$	1	Steady-state population of macrophages
*n*	1	Hill-coefficient like parameter
*e*	2	Modulatory effect of keratinocytes on other modeled cell types and itself
*f*	0.5	Modulatory effect of dendritic cells on other modeled cell types and itself

### 2.4 Sensitivity analysis

We performed a global sensitivity analysis using Sobol GSA Software (Kucherenko and Zaccheus, 2024) with MATLAB R2022b. Sobol’s method (Sobol, 1993) of sensitivity analysis quantifies the relative importance of input parameters by decomposing the total variance into contributions from individual parameters as well as combinations of parameters. Default sample values were generated using Sobol sequences according to parameter ranges, and their distributions are defined in Table 3. During this analysis, the software samples the parameter space simultaneously, considering all the parameters together, and reports two types of sensitivity index: first-order and total sensitivity. The first-order sensitivity indices are used to show the fractional contribution of a single parameter to the output variance, while higher-order sensitivity indices are calculated to measure the fractional contribution of parameter interactions along with the fractional contribution of individual model parameters to the output variance. In our results, we report the first-order sensitivity index to show the contribution of each model parameter to the total model output variance. Therefore, all model parameters are varied simultaneously in this approach, rather than one parameter at a time. The parameter ranges are chosen based on the observation that significant changes in the keratinocyte population were observed in the given range of parameters.

**TABLE 3:** Parameters, their range of variation, and assumed distribution for global sensitivity analysis.

Parameters	Range	Distribution
$\beta_{IL36}^{\prime }$	0–10 days^−1^	Uniform
$\bar{M}$	0–10	Uniform
*n*	0–10	Uniform
*e*	0–10	Uniform
*f*	0–10	Uniform

### 2.5 Bifurcation analysis

A bifurcation analysis is performed to investigate the steady-state behavior of the model and how it changes when parameter values are varied using an open-access tool, MATCONT (Govaerts et al., 2008), and MATLAB software (The MathWorks, Inc., 2022).

## 3 Results

We investigate the steady-state behavior of the network mediated by IL-36 (Figure 1) using a standard bifurcation analysis technique of dynamical systems theory. IL-36 is a proinflammatory cytokine, and its role in the progression of psoriasis has been observed (Mahil et al., 2017). Here, we study the effect of changes in the level of IL-36 cytokine on the population of keratinocytes for varying model parameters.

**FIGURE 2:**
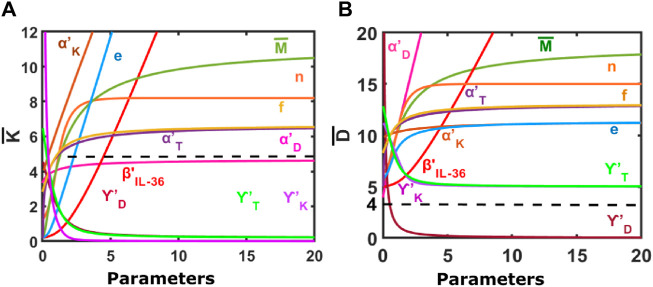
Steady-state response of the model to changes in different model parameters. **(A)** Change in the population of keratinocytes in response to varying model parameters. **(B)** Change in the population of dendritic cells in response to varying model parameters. Default parameters are: $\alpha_{K}^{\prime }$ = 0.1 day^−1^, β_IL−36_ = 4 days^−1^, n = 1, $\bar{M}$ = 1, γ_K_ = 0.5 days^−1^, γ_D_ = 0.2 days^−1^, γ_T_ = 0.5 days^−1^, $\alpha_{D}^{\prime }$ = 1 day^−1^, e =2, $\alpha_{T}^{\prime }$ = 0.5 days^−1^, f = 0.5. The initial condition is $\bar{K}=4,\bar{D}=2,\bar{T}=0.1$ The dashed line in the figure serves as a threshold for the keratinocyte population. Keratinocytes above this threshold are believed to indicate the psoriatic state (Valeyev et al., 2010).

Our results suggest that an increase in the population of K and D would be observed for an increase in the level of the IL-36 (represented by $\beta_{IL-36}^{\prime }$ ) (Figure 2). A four-fold increase in the keratinocyte population is believed to be an indicator of a psoriatic condition, based on the observation by Valeyev et al. (2010). Therefore, a significant increase in the level of IL-36 would lead to a psoriatic state, and increased IL-36 levels have been observed in psoriasis (Mahil et al., 2017). In addition, a significant increase in the dendritic cell population has been observed in the pustule formation stage of GPP (Vyas et al., 2019). We observed that an increase in the value of the parameter *e* ( $e=\frac{k_{D}}{k_{K}}$ ) would cause a sharp increase in the keratinocyte population, indicating the modulatory effect of keratinocytes on the other modeled cell types is stronger than the modulatory effect of dendritic cells, whereas a gradual increase in the dendritic cell population is observed for an increase in the values of parameter *e* (Figures 2A, B), which saturates for large values of *e*.

In addition, the K and D populations would increase in the psoriatic lesion with an increase in the effective rate of migration of keratinocytes, dendritic cells, T cells, and macrophages (represented by $\alpha_{K}^{\prime }$ , $\alpha_{D}^{\prime }$ , $\alpha_{T}^{\prime }$ , and $\alpha_{\bar{M}}^{\prime }$ , respectively), as shown in Figure 2A.

Furthermore, an increase in the values of parameter *f* $\left(f=\frac{k_{D}}{k_{T}}\right)$ would result in a gradual increase in the population of K and D, which saturates for further increase in *f* (Figures 2A, B). This suggests that the modulatory effect of the dendritic cell population on other modeled cells and itself is weak compared to that of T cells.

Our results also suggest an increase in the Hill-coefficient-like parameter *n* would lead to an initial increase in the keratinocyte and dendritic cell populations that saturate at large values of *n*.

For an increase in the steady-state population of macrophages $\left(\bar{M}\right)$ , the populations of keratinocyte and dendritic cells both increase gradually (Figure 2).

**FIGURE 3:**
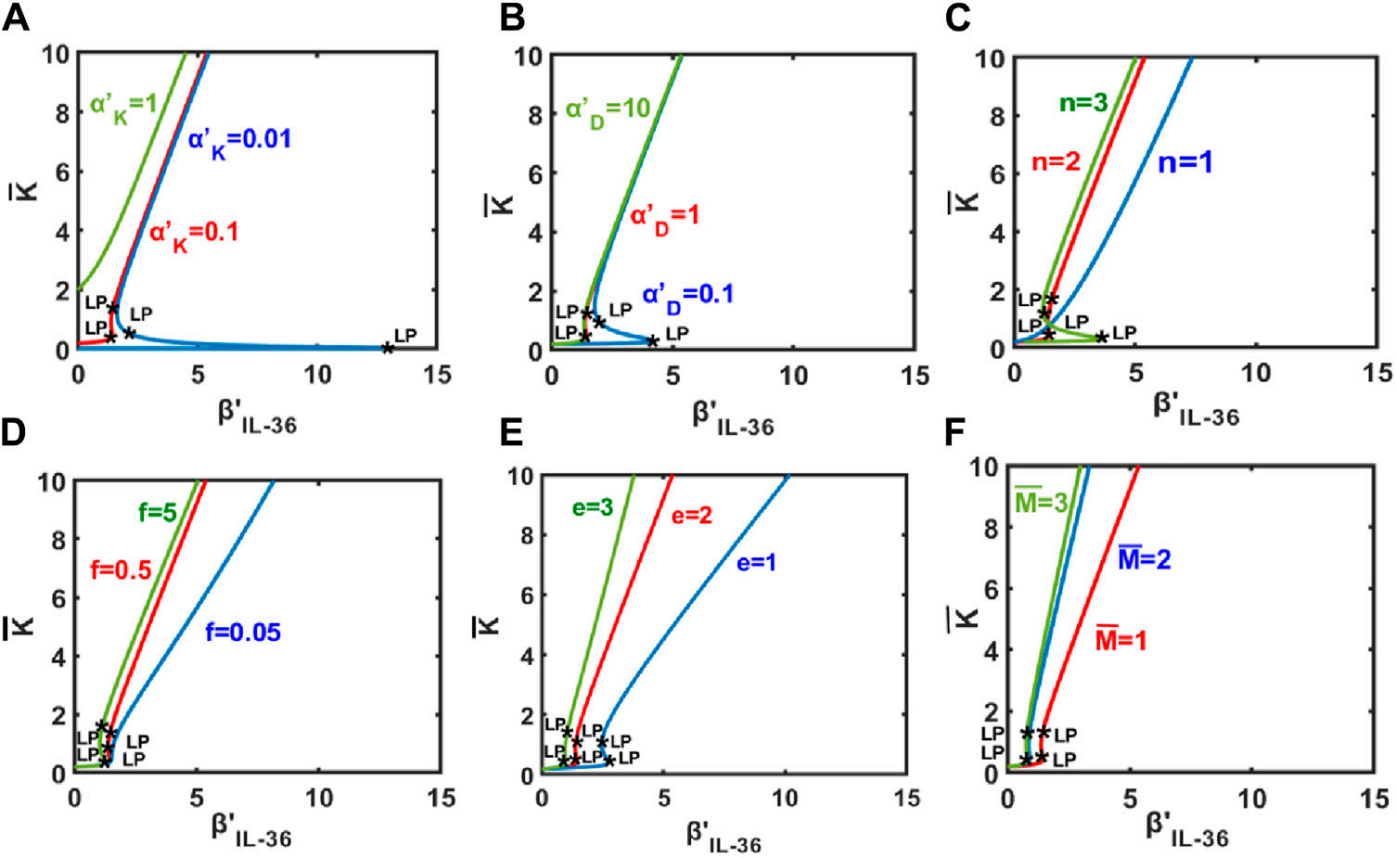
Keratinocyte population as a function of the level of IL-36 denoted by β′_IL−36_. Change in keratinocyte population as a function of the level of IL-36 for different values of **(A)** $\alpha_{K}^{\prime }$ , **(B)** $\alpha_{D}^{\prime }$ , **(C)** n,**(D)** f, **(E)** e, and **(F)** $\bar{M}$ . Default parameter values are $\alpha_{K}^{\prime }$ = 0.1 day^−1^, β_IL−36_ = 4 days^−1^, n = 2, $\bar{M}=1$ , γ_K_ = 0.5 days^−1^, γ_D_ = 0.2 days^−1^, γ_T_ = 0.5 days^−1^, $\alpha_{D}^{\prime }$ = 1 day^−1^, e = 2, and $\alpha_{T}^{\prime }$ = 0.5 days^−1^, and f = 0.5. The initial condition is $\bar{K}=4,\bar{D}=2,\bar{T}=0.1$ .

Through Figure 3, we demonstrate how the change in the population of keratinocytes upon varying the level of IL-36 (represented by *β*_*IL*−36_) depends on other model parameters. Our model results suggest a sharp increase in the keratinocyte population for an increase in the effective rate of inward migration of keratinocytes $\left(\alpha_{K}^{\prime }\right)$, as depicted in Figure 3A. A switch-like increase would be observed for small values of $\alpha_{K}^{\prime }$ in Figure 3A. The bistable region bounded by the limit points would decrease with the increase in the $\alpha_{K}^{\prime }$ . A sharp increase in the keratinocyte population would also be observed for increases in the effective rate of inward migration of dendritic cells $\left(\alpha_{D}^{\prime }\right)$ and the effective rate of inward migration of T cells $\left(\alpha_{T}^{\prime }\right)$ (Figure 3B and Supplementary Figure S1). In the case of a switch-like increase in the population of keratinocytes, the bistable region would decrease with an increase in $\alpha_{D}^{\prime }$ and $\alpha_{T}^{\prime }$ (Figure 3B and Supplementary Figure S1).

An increase in the keratinocyte population would also be observed for an increase in the Hill-coefficient like parameter *n* (Figure 3C). For a small value of *n*, the population of keratinocytes would increase gradually. A switch-like increase is expected for a large value of *n*, and the bistable region would be larger for *n* = 3 than for *n* = 2.

Our results suggest gradual and switch-like increases in the population of keratinocytes for an increase in values of *f* (denoting the modulatory effect of keratinocytes on itself) and *e* (denoting the modulatory effect of dendritic cells on other modeled cell types, respectively (Figures 3D, E). Similar observations would be expected for an increase in the steady-state population of macrophages $\left(\bar{M}\right)$ (Figure 3F).

### 3.1 Based on sensitivity analysis

**FIGURE 4:**
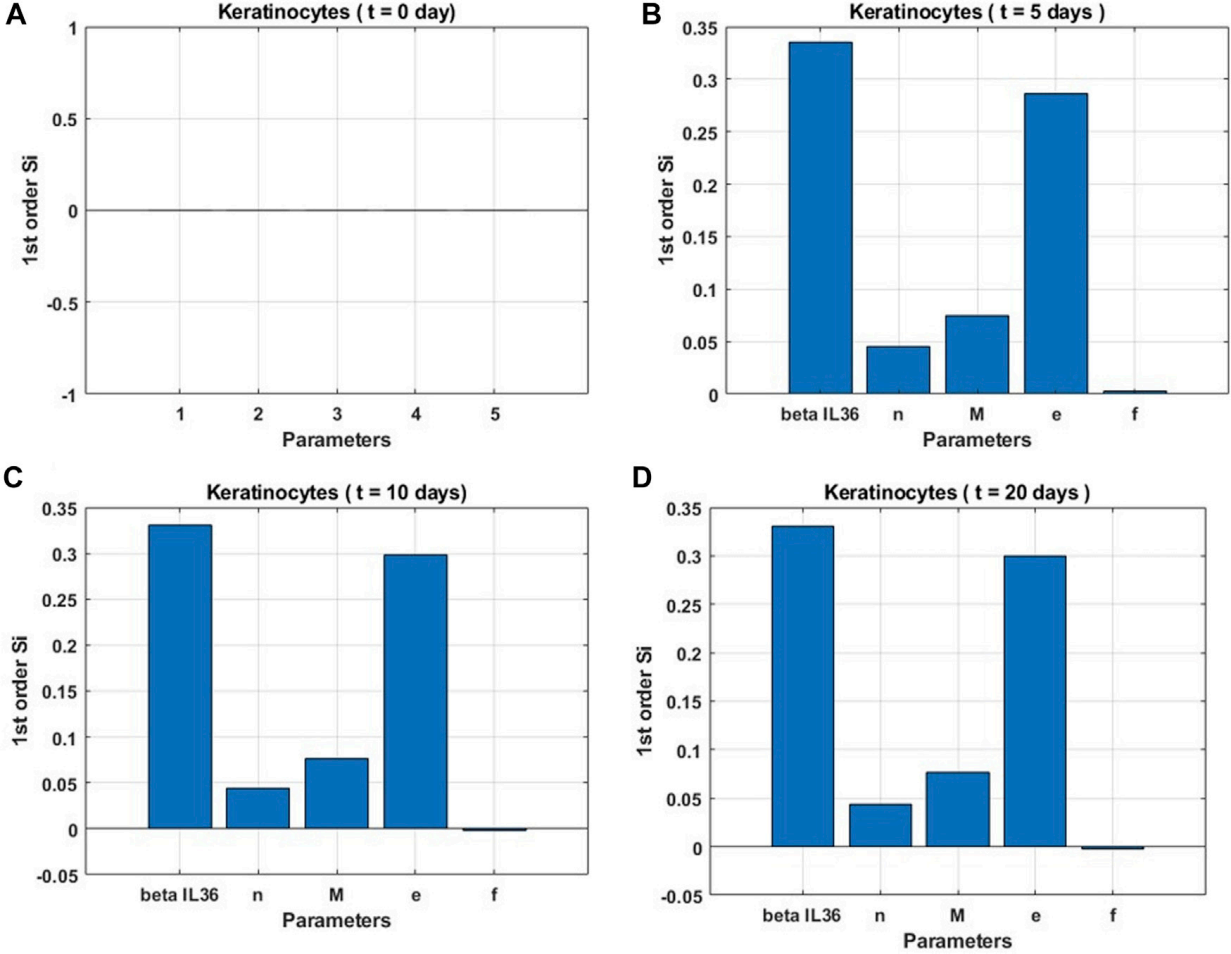
Global sensitivity indices for keratinocyte population. **(A)** at the start (t = 0 days), **(B)** before the onset of steady state (t = 5 days), **(C)** at the onset of steady state (t = 10 days), and **(D)** post the onset of steady state (t = 20 days).

Sensitivity indices for five parameters were obtained to assess their impact on the rate of change in the keratinocyte population (Figure 4) at different time points in the process of attaining the steady state. Our analysis suggests that the change in the parameter *β*_*IL*−36_, which represents the level of IL-36 cytokines, was most influential over all time points for the change in keratinocyte cell population followed by the modulatory effects of keratinocytes on other cell populations and itself (e), the population of macrophages $\left(\bar{M}\right)$ , and Hill-coefficient like parameter (*n*), respectively. In addition, we observed that the change in keratinocyte population was unaffected by the change in modulatory effects of dendritic cells over the other cell population and itself (f).

### 3.2 Based on bifurcation analysis

**FIGURE 5:**
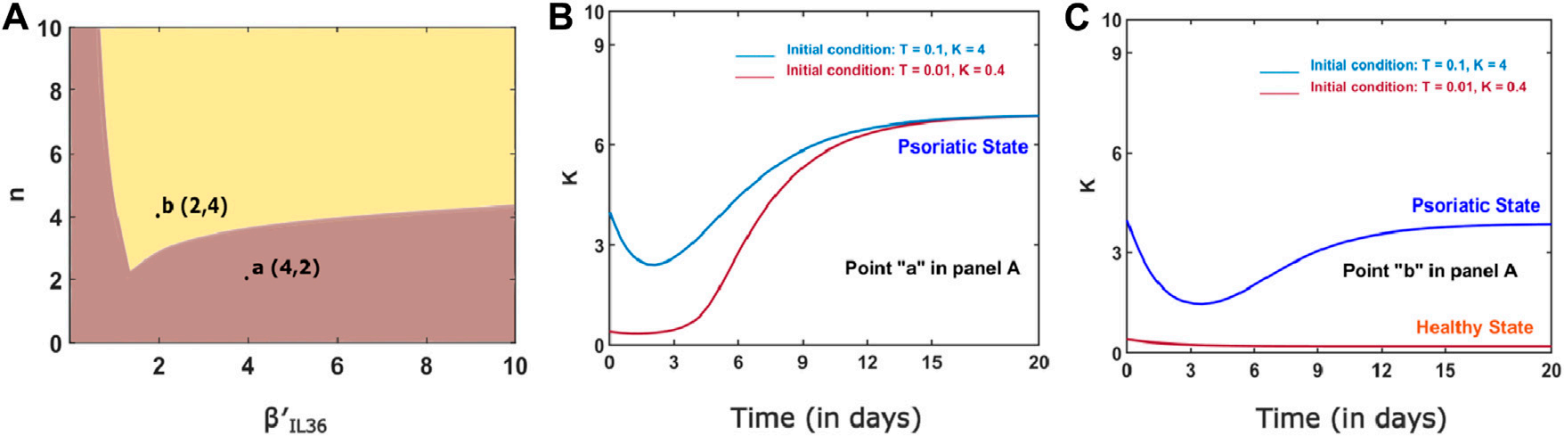
**(A)** Two-parameter bifurcation curve for n and $\beta_{IL-36}^{\prime }$ . **(B)** Dynamics of the network for a model parameter combination that lies inside a bistable region, point b in Panel A of Figure 5. **(C)** Dynamics of the network for a model parameter combination that lies inside a monostable region at point a in Panel **A**. The values of parameters and initial condition are the same as in Figure 3.

The switch-like increase in keratinocytes would be a robust behavior as we found large bistable regions in two-parameter space spanned by (*n*, $\beta_{IL-36}^{\prime }$ ) (Figure 5A). A typical temporal behavior of the network in bistable and monostable regions is shown in Figures 5B, C.

## 4 Discussion

Psoriasis is characterized by the formation of a psoriatic lesion that is infiltrated by immune cell populations (Lowes et al., 2014; Dietrich et al., 2016). Several mathematical models have been developed to elucidate the role of cytokines in the progression of psoriasis (Roy et al., 2010; Valeyev et al., 2010; Roy and Datta, 2012). One of the models explored the roles of IL-15, IL-17/IL-23, and TNF*α* cytokines mediating indirect cell–cell interactions among keratinocytes, dendritic cells, and T cells in a psoriatic lesion (Pandey et al., 2021). These cytokines have been targeted in earlier studies that demonstrate their clinical potential.

Different types of psoriasis have been reported, and psoriasis vulgaris is the most prevalent type. GPP is another severe form of psoriasis. In GPP, relapsing sterile pustules are present in the affected individuals (Samotij et al., 2021). Recent studies attribute this relapse to T cells (skin resident memory TRM cell, and memory like *γδ*T cells) whose proliferation and differentiation are caused by dendritic cells through a feed-forward loop involving inflammatory cytokines (reviewed in (Tiang and Lai, 2022)). The dermal dendritic cells colocalize with neutrophils that secrete elastase, which is believed to have a role in pustule formation (Skrzeczynska-Moncznik et al., 2012). Studies have shown that the elevated presence of human neutrophil elastase in GPP contributes to tissue damage, degradation of extracellular matrix, and disruption of the skin’s barrier function (Johnston et al., 2017). Hence, an increase in DC levels and their indirect interaction with elastase (Wittamer et al., 2005) could lead to worsening of psoriasis. Additionally, neutrophil elastase activates the cytokine IL-36*γ*, resulting in the exacerbation of psoriasis (Clancy et al., 2017).

Recent studies have unveiled IL-36 as a potential drug target treatment of GPP/psoriasis (Bachelez et al., 2019; Choon et al., 2021a; Choon et al., 2021b). This hypothesis is augmented by gene expression data that report a significant upregulation of the IL-36 cluster of cytokines in psoriasis patients (D’erme et al., 2015). To explore the role of proinflammatory cytokine IL-36 in the plaque formation and disease progression of psoriasis, we have built a network of indirect cell–cell interactions among key immune cells (dendritic cells, macrophages, and T-lymphocytes) and skin cells (keratinocytes) mediated by IL-36. Our results are consistent with the finding that IL-36 could lead to worsening of psoriasis. Furthermore, a sensitivity analysis for parameters (in Table 3) affecting the function of the network demonstrates that the cell populations are most sensitive to the level of IL-36. Our results suggest an increase in the population of keratinocytes; thus, the plaque formation and progression of psoriasis could occur either in a gradual or a switch-like manner. The switch-like-on set or progression of psoriasis would occur due to the bistable behavior of the network due to the indirect immune and keratinocyte cell interactions via IL-36. An exploration of the parameters controlling the bistable region suggests the switch-like abrupt changes in the keratinocyte population are a robust phenomenon. For instance, we found a sizeable bistable region in a two-parameter space spanned by the level of IL-36 $\left(\beta_{IL-36}^{\prime }\right)$ vs. the Hill-coefficient-like parameter *n*).

One limitation of our model is that it is formulated for only a single cytokine, whereas the role of several cytokines in psoriasis has been well-reported. Here, it is assumed that although several cytokines are involved in psoriasis, IL-36 has a dominant role, especially in GPP. Another limitation of the model is that all the IL-36 agonists (*α*, *β*, and *γ*) are represented as one single cytokine IL-36 instead of considering its isoforms separately. A future model could consider all the isoforms of the cytokine IL36, *α*, *β*, and *γ*, separately, and the role of different isoforms of IL-36 in the pathogenesis of psoriasis can be explored systematically. In addition, model results are yet to be experimentally verified. However, the present framework could also be used to study the role of a dominant cytokine in other inflammatory and autoimmune diseases.

This study highlights the network dynamics approach toward understanding the pathogenesis of psoriasis, which is a novel way to study cell–cell interactions in the context of human physiology. Several other studies have applied network dynamics to interpret organ interactions (Ivanov et al., 2021) and cortico-muscular interactions (Rizzo et al., 2020) and lay the groundwork in the field of network physiology and network medicine (Ivanov et al., 2016). Other models use fractional order differential equations to investigate the phenomenon of memory trace and study the influence of immune-boosting drugs on psoriasis (Özköse, 2024). This broadens the conventional approach of disease models framed by linear ODEs to include those featuring fractional order.

## Data Availability

The original contributions presented in the study are included in the article/Supplementary Material; further inquiries can be directed to the corresponding author.
