# Supplementary material for: Dynamics of a network mediated by IL-36 and involved in the pathogenesis of psoriasis
Source: Front Netw Physiol. 2024 May 31;4:1363791. doi: 10.3389/fnetp.2024.1363791 (PMC11176455; doi:10.3389/fnetp.2024.1363791)
Supplement: Supplementary file 1 [file DataSheet2.pdf]

Supplementary Analysis: Boundedness, Existence and Uniqueness Analysis of the model

Dynamics of the Network Mediated by IL-36 in the Pathogenesis of Psoriasis

Sneha Pandey[1,*], Syona Tiwari[1,*], Sulagna Basu[1,*], Rajiv Kumar Mishra[2] and Rakesh Pandey[1,++]
[1] Bioinformatics, MMV, Banaras Hindu University, Varanasi, Uttar Pradesh, India
[2] iOligos Technologies Private Limited, India

[*] *Contributed equally*
[++] *Correspondence: rakeshpandey@bhu.ac.in*

Original ODE System

The original ordinary differential equation (ODE) system is given by:

$$\frac{d\bar{D}}{dt} = \alpha\prime_D + \beta\prime_{IL36}\left(\frac{\bar{D}^n}{1+\bar{D}^n}\frac{\bar{T}^n}{1+\bar{T}^n}\frac{\bar{M}^n}{1+\bar{M}^n}\frac{\bar{K}^n}{1+\bar{K}^n}\right) - \gamma_D\bar{D}$$

$$\frac{d\bar{K}}{dt} = \alpha\prime_K + \beta\prime_{IL36}\cdot e\left(\frac{\bar{D}^n}{1+\bar{D}^n}\frac{\bar{T}^n}{1+\bar{T}^n}\frac{\bar{M}^n}{1+\bar{M}^n}\frac{\bar{K}^n}{1+\bar{K}^n}\right) - \gamma_K\bar{K}$$

$$\frac{d\bar{T}}{dt} = \alpha\prime_T + \beta\prime_{IL36}\cdot f\left(\frac{\bar{D}^n}{1+\bar{D}^n}\frac{\bar{T}^n}{1+\bar{T}^n}\frac{\bar{M}^n}{1+\bar{M}^n}\frac{\bar{K}^n}{1+\bar{K}^n}\right) - \gamma_T\bar{T}$$

Where:

- $\alpha\prime_D, \alpha\prime_K, \alpha\prime_T, \beta\prime_{IL36}, e, f, \gamma_D, \gamma_K, \gamma_T$ are positive constants representing parameters.
- $\bar{D}, \bar{K}, \bar{T}, \text{and}\bar{M}$represent the population of modelled cells. They will have $\bar{D}_0, \bar{K}_0, \bar{T}_0, \text{and}\bar{M}_0$ initial conditions which are positive too.

1 Analysis of Positivity

Since all the state variables are representing no. of cells therefore they will always remmain positive.

2 Analysis of Boundedness

We analyze the boundedness of the system's RHS, and found that systems is bounded provided following conditions are satisfied

1. For the first equation:
$$\left|\frac{d\bar{D}}{dt}\right| \leq \left|\alpha'\prime_D + \beta'\prime_{IL36} - \gamma_D\bar{D}\right|$$
2. For the second equation:
$$\left|\frac{d\bar{K}}{dt}\right| \leq \left|\alpha'\prime_K + \beta'\prime_{IL36}\cdot e - \gamma_K\bar{K}\right|$$

3. For the third equation:

$$\left|\frac{\mathrm{d}\bar{T}}{\mathrm{d}t}\right| \leq \left|\alpha'{\prime}_T + \beta'{\prime}_{IL36} \cdot f - \gamma_T \bar{T}\right|$$

These inequalities ensure that the derivatives of the state variables remain bounded by the corresponding expressions on the right-hand side of the equations, facilitating a comprehensive understanding of the system's boundedness.

3 Analysis of Existence and Uniqueness

Assume that a vector field $\mathbf{F}(\bar{D}, \bar{K}, \bar{T})$ defined as following represents the RHS of the ODE system

$$\mathbf{F}(\bar{D}, \bar{K}, \bar{T}) = \begin{pmatrix} \alpha\prime_D + \beta\prime_{IL36}\left(\frac{\bar{D}^n}{1+\bar{D}^n}\frac{\bar{T}^n}{1+\bar{T}^n}\frac{\bar{M}^n}{1+\bar{M}^n}\frac{\bar{K}^n}{1+\bar{K}^n}\right) - \gamma_D \bar{D} \\ \alpha\prime_K + \beta\prime_{IL36} \cdot e\left(\frac{\bar{D}^n}{1+\bar{D}^n}\frac{\bar{T}^n}{1+\bar{T}^n}\frac{\bar{M}^n}{1+\bar{M}^n}\frac{\bar{K}^n}{1+\bar{K}^n}\right) - \gamma_K \bar{K} \\ \alpha\prime_T + \beta\prime_{IL36} \cdot f\left(\frac{\bar{D}^n}{1+\bar{D}^n}\frac{\bar{T}^n}{1+\bar{T}^n}\frac{\bar{M}^n}{1+\bar{M}^n}\frac{\bar{K}^n}{1+\bar{K}^n}\right) - \gamma_T \bar{T} \end{pmatrix}$$

where $\alpha\prime_D$, $\alpha\prime_K$, $\alpha\prime_T$, $\beta\prime_{IL36}$, e, f, γ_D, γ_K, γ_T are positive constants.

3.1 Analysis of Continuity of Vector Field

To ensure the continuity of the vector field $\mathbf{F}(\bar{D}, \bar{K}, \bar{T})$, we examine each term in $\mathbf{F}$ and verify its continuity with respect to $\bar{D}$, $\bar{K}$, and $\bar{T}$, we need to ensure that the denominators $1+\bar{D}^n$, $1+\bar{K}^n$, and $1+\bar{T}^n$ are always positive to avoid division by zero. The functions $\frac{\bar{D}^n}{1+\bar{D}^n}$, $\frac{\bar{K}^n}{1+\bar{K}^n}$, and $\frac{\bar{T}^n}{1+\bar{T}^n}$ are continuous for all positive values of $\bar{D}$, $\bar{K}$, and $\bar{T}$.

Since the denominators are always positive and the functions are rational functions with continuous numerator and denominator for all positive values of $\bar{D}$, $\bar{K}$, and $\bar{T}$, we conclude that $\mathbf{F}(\bar{D}, \bar{K}, \bar{T})$ is continuous everywhere for positive values of $\bar{D}$, $\bar{K}$, and $\bar{T}$.

3.2 Partial derivatives and continuity of them

$\mathbf{F}(\bar{D}, \bar{K}, \bar{T})$ as a vector composed of three functions $F_1(\bar{D}, \bar{K}, \bar{T})$, $F_2(\bar{D}, \bar{K}, \bar{T})$, and $F_3(\bar{D}, \bar{K}, \bar{T})$:

$$\mathbf{F}(\bar{D}, \bar{K}, \bar{T}) = \begin{pmatrix} F_1(\bar{D}, \bar{K}, \bar{T}) \\ F_2(\bar{D}, \bar{K}, \bar{T}) \\ F_3(\bar{D}, \bar{K}, \bar{T}) \end{pmatrix}$$

Where each component F_i for $i = 1, 2, 3$ is defined as follows:

1. $F_1(\bar{D}, \bar{K}, \bar{T})$:

$$F_1(\bar{D}, \bar{K}, \bar{T}) = \alpha\prime_D + \beta\prime_{IL36}\left(\frac{\bar{D}^n}{1+\bar{D}^n}\frac{\bar{T}^n}{1+\bar{T}^n}\frac{\bar{M}^n}{1+\bar{M}^n}\frac{\bar{K}^n}{1+\bar{K}^n}\right) - \gamma_D \bar{D}$$

2. $F_2(\bar{D}, \bar{K}, \bar{T})$:

$$F_2(\bar{D}, \bar{K}, \bar{T}) = \alpha\prime_K + \beta\prime_{IL36} \cdot e\left(\frac{\bar{D}^n}{1+\bar{D}^n}\frac{\bar{T}^n}{1+\bar{T}^n}\frac{\bar{M}^n}{1+\bar{M}^n}\frac{\bar{K}^n}{1+\bar{K}^n}\right) - \gamma_K \bar{K}$$

3. $F_3(\bar{D}, \bar{K}, \bar{T})$:

$$F_3(\bar{D}, \bar{K}, \bar{T}) = \alpha\prime_T + \beta\prime_{IL36} \cdot f\left(\frac{\bar{D}^n}{1+\bar{D}^n}\frac{\bar{T}^n}{1+\bar{T}^n}\frac{\bar{M}^n}{1+\bar{M}^n}\frac{\bar{K}^n}{1+\bar{K}^n}\right) - \gamma_T \bar{T}$$

Now lets compute the partial derivatives of $\mathbf{F}$.

Partial derivatives of F with respect to $\bar{D}$:

$$\frac{\partial F}{\partial \bar{D}} = \begin{bmatrix} \frac{\partial F_1}{\partial \bar{D}} & \frac{\partial F_2}{\partial \bar{D}} & \frac{\partial F_3}{\partial \bar{D}} \end{bmatrix}$$

Partial derivatives of F with respect to $\bar{K}$:

$$\frac{\partial F}{\partial \bar{K}} = \begin{bmatrix} \frac{\partial F_1}{\partial \bar{K}} & \frac{\partial F_2}{\partial \bar{K}} & \frac{\partial F_3}{\partial \bar{K}} \end{bmatrix}$$

Partial derivatives of F with respect to $\bar{T}$:

$$\frac{\partial F}{\partial \bar{T}} = \begin{bmatrix} \frac{\partial F_1}{\partial \bar{T}} & \frac{\partial F_2}{\partial \bar{T}} & \frac{\partial F_3}{\partial \bar{T}} \end{bmatrix}$$

Partial derivatives of F_1:

$$\begin{aligned}
\frac{\partial F_1}{\partial \bar{D}} &= \beta\prime_{IL36}\left(\frac{n\bar{D}^{n-1}}{(1+\bar{D}^n)^2}\frac{\bar{T}^n}{1+\bar{T}^n}\frac{\bar{M}^n}{1+\bar{M}^n}\frac{\bar{K}^n}{1+\bar{K}^n}\right) - \gamma_D \\
\frac{\partial F_1}{\partial \bar{K}} &= \beta\prime_{IL36}\left(\frac{\bar{D}^n}{1+\bar{D}^n}\frac{\bar{T}^n}{1+\bar{T}^n}\frac{\bar{M}^n}{1+\bar{M}^n}\right)\left(\frac{n\bar{K}^{n-1}}{(1+\bar{K}^n)^2}\right) \\
\frac{\partial F_1}{\partial \bar{T}} &= \beta\prime_{IL36}\left(\frac{\bar{D}^n}{1+\bar{D}^n}\frac{\bar{M}^n}{1+\bar{M}^n}\frac{\bar{K}^n}{1+\bar{K}^n}\right)\left(\frac{n\bar{T}^{n-1}}{(1+\bar{T}^n)^2}\right)
\end{aligned}$$

Partial derivatives of F_2:

$$\begin{aligned}
\frac{\partial F_2}{\partial \bar{D}} &= \beta\prime_{IL36}\cdot e\left(\frac{n\bar{D}^{n-1}}{(1+\bar{D}^n)^2}\frac{\bar{T}^n}{1+\bar{T}^n}\frac{\bar{M}^n}{1+\bar{M}^n}\frac{\bar{K}^n}{1+\bar{K}^n}\right) \\
\frac{\partial F_2}{\partial \bar{K}} &= -\gamma_K + \beta\prime_{IL36}\cdot e\left(\frac{\bar{D}^n}{1+\bar{D}^n}\frac{\bar{T}^n}{1+\bar{T}^n}\frac{\bar{M}^n}{1+\bar{M}^n}\right)\left(\frac{n\bar{K}^{n-1}}{(1+\bar{K}^n)^2}\right) \\
\frac{\partial F_2}{\partial \bar{T}} &= \beta\prime_{IL36}\cdot e\left(\frac{\bar{D}^n}{1+\bar{D}^n}\frac{\bar{M}^n}{1+\bar{M}^n}\frac{\bar{K}^n}{1+\bar{K}^n}\right)\left(\frac{n\bar{T}^{n-1}}{(1+\bar{T}^n)^2}\right)
\end{aligned}$$

Partial derivatives of F_3:

$$\begin{aligned}
\frac{\partial F_3}{\partial \bar{D}} &= \beta\prime_{IL36}\cdot f\left(\frac{n\bar{D}^{n-1}}{(1+\bar{D}^n)^2}\frac{\bar{T}^n}{1+\bar{T}^n}\frac{\bar{M}^n}{1+\bar{M}^n}\frac{\bar{K}^n}{1+\bar{K}^n}\right) \\
\frac{\partial F_3}{\partial \bar{K}} &= \beta\prime_{IL36}\cdot f\left(\frac{\bar{D}^n}{1+\bar{D}^n}\frac{\bar{T}^n}{1+\bar{T}^n}\frac{\bar{M}^n}{1+\bar{M}^n}\right)\left(\frac{n\bar{K}^{n-1}}{(1+\bar{K}^n)^2}\right) \\
\frac{\partial F_3}{\partial \bar{T}} &= -\gamma_T + \beta\prime_{IL36}\cdot f\left(\frac{\bar{D}^n}{1+\bar{D}^n}\frac{\bar{M}^n}{1+\bar{M}^n}\frac{\bar{K}^n}{1+\bar{K}^n}\right)\left(\frac{n\bar{T}^{n-1}}{(1+\bar{T}^n)^2}\right)
\end{aligned}$$

We can summarize and rewrite the partial derivatives of F with respect to $\bar{D}$, $\bar{K}$, and $\bar{T}$ in a more concise manner:

$$\begin{aligned}
&\begin{bmatrix} \frac{\partial F_1}{\partial \bar{D}} & \frac{\partial F_1}{\partial \bar{K}} & \frac{\partial F_1}{\partial \bar{T}} \end{bmatrix} \\
&= \beta\prime_{IL36}\left[\left(\frac{n\bar{D}^{n-1}}{(1+\bar{D}^n)^2}\frac{\bar{T}^n}{1+\bar{T}^n}\frac{\bar{M}^n}{1+\bar{M}^n}\frac{\bar{K}^n}{1+\bar{K}^n}\right) \quad \left(\frac{\bar{D}^n}{1+\bar{D}^n}\frac{\bar{T}^n}{1+\bar{T}^n}\frac{\bar{M}^n}{1+\bar{M}^n}\right)\left(\frac{n\bar{K}^{n-1}}{(1+\bar{K}^n)^2}\right)\left(\frac{\bar{D}^n}{1+\bar{D}^n}\frac{\bar{M}^n}{1+\bar{M}^n}\frac{\bar{K}^n}{1+\bar{K}^n}\right)\left(\frac{n\bar{T}^{n-1}}{(1+\bar{T}^n)^2}\right)\right]
\end{aligned}$$

$$\begin{aligned}
&\begin{bmatrix} \frac{\partial F_2}{\partial \bar{D}} & \frac{\partial F_2}{\partial \bar{K}} & \frac{\partial F_2}{\partial \bar{T}} \end{bmatrix} \\
&= \beta\prime_{IL36}\cdot e\left[\left(\frac{n\bar{D}^{n-1}}{(1+\bar{D}^n)^2}\frac{\bar{T}^n}{1+\bar{T}^n}\frac{\bar{M}^n}{1+\bar{M}^n}\frac{\bar{K}^n}{1+\bar{K}^n}\right) \quad -\gamma_K + \left(\frac{\bar{D}^n}{1+\bar{D}^n}\frac{\bar{T}^n}{1+\bar{T}^n}\frac{\bar{M}^n}{1+\bar{M}^n}\right)\left(\frac{n\bar{K}^{n-1}}{(1+\bar{K}^n)^2}\right) \quad \left(\frac{\bar{D}^n}{1+\bar{D}^n}\frac{\bar{M}^n}{1+\bar{M}^n}\frac{\bar{K}^n}{1+\bar{K}^n}\right)\left(\frac{n\bar{T}^{n-1}}{(1+\bar{T}^n)^2}\right)\right]
\end{aligned}$$

$$\begin{aligned}
&\begin{bmatrix} \frac{\partial F_3}{\partial \bar{D}} & \frac{\partial F_3}{\partial \bar{K}} & \frac{\partial F_3}{\partial \bar{T}} \end{bmatrix} \\
&= \beta\prime_{IL36}\cdot f\left[\left(\frac{n\bar{D}^{n-1}}{(1+\bar{D}^n)^2}\frac{\bar{T}^n}{1+\bar{T}^n}\frac{\bar{M}^n}{1+\bar{M}^n}\frac{\bar{K}^n}{1+\bar{K}^n}\right) \quad \left(\frac{\bar{D}^n}{1+\bar{D}^n}\frac{\bar{T}^n}{1+\bar{T}^n}\frac{\bar{M}^n}{1+\bar{M}^n}\right)\left(\frac{n\bar{K}^{n-1}}{(1+\bar{K}^n)^2}\right) \quad -\gamma_T + \left(\frac{\bar{D}^n}{1+\bar{D}^n}\frac{\bar{M}^n}{1+\bar{M}^n}\frac{\bar{K}^n}{1+\bar{K}^n}\right)\left(\frac{n\bar{T}^{n-1}}{(1+\bar{T}^n)^2}\right)\right]
\end{aligned}$$

To ensure the continuity of the partial derivatives of F with respect to $\bar{D}$, $\bar{K}$, and $\bar{T}$, we need to ensure that the expressions for these partial derivatives are continuous functions of their respective variables. Specifically, we need to ensure that each term in the expressions remains continuous for all values of $\bar{D}$, $\bar{K}$, and $\bar{T}$ greater than zero.

Let's examine each partial derivative individually:

1. For $\frac{\partial F}{\partial \bar{D}}$: - All terms involving $\bar{D}$, $\bar{K}$, and $\bar{T}$ are continuous functions. - The denominator terms $(1+\bar{D}^n)^2$, $(1+\bar{K}^n)^2$, and $(1+\bar{T}^n)^2$ are always positive for $\bar{D}$, $\bar{K}$, and $\bar{T}$ greater than zero. - Therefore, the partial derivative $\frac{\partial F}{\partial \bar{D}}$ is continuous.

2. For $\frac{\partial F}{\partial \bar{K}}$: - Similar to the previous case, all terms involving $\bar{D}$, $\bar{K}$, and $\bar{T}$ are continuous functions. - The denominator terms $(1+\bar{D}^n)^2$, $(1+\bar{K}^n)^2$, and $(1+\bar{T}^n)^2$ are always positive for $\bar{D}$, $\bar{K}$, and $\bar{T}$ greater than zero. - Hence, the partial derivative $\frac{\partial F}{\partial \bar{K}}$ is continuous.

3. For $\frac{\partial F}{\partial \bar{T}}$: - Once again, all terms involving $\bar{D}$, $\bar{K}$, and $\bar{T}$ are continuous functions. - The denominator terms $(1+\bar{D}^n)^2$, $(1+\bar{K}^n)^2$, and $(1+\bar{T}^n)^2$ are always positive for $\bar{D}$, $\bar{K}$, and $\bar{T}$ greater than zero. - Consequently, the partial derivative $\frac{\partial F}{\partial \bar{T}}$ is continuous.

Therefore, the continuity condition for the partial derivatives of F with respect to $\bar{D}$, $\bar{K}$, and $\bar{T}$ is satisfied.

Together the above analysis shows that our ODE systems satisfies the Existence and Uniqueness theorem of ODE. So, we can conclude that our model system will always have a uniquene solution for a set of parameters and an initial condition.
