# Supplementary material for: Dynamics of a network mediated by IL-36 and involved in the pathogenesis of psoriasis
Source: Front Netw Physiol. 2024 May 31;4:1363791. doi: 10.3389/fnetp.2024.1363791 (PMC11176455; doi:10.3389/fnetp.2024.1363791)
Supplement: Supplementary file 2 [file DataSheet1.PDF]

Supporting Information

Dynamics of the Network Mediated by IL-36 in the Pathogenesis of Psoriasis

Sneha Pandey[1, *], Syona Tiwari[1, *], Sulagna Basu[1, *], Rajiv Kumar Mishra[2], and Rakesh Pandey[1, ++]

[1] Bioinformatics, MMV, Banaras Hindu University, Varanasi, Uttar Pradesh, India
[2] iOligos Technologies Private Limited, India

*Contributed equally
++ Correspondance: rakeshpandey@bhu.ac.in

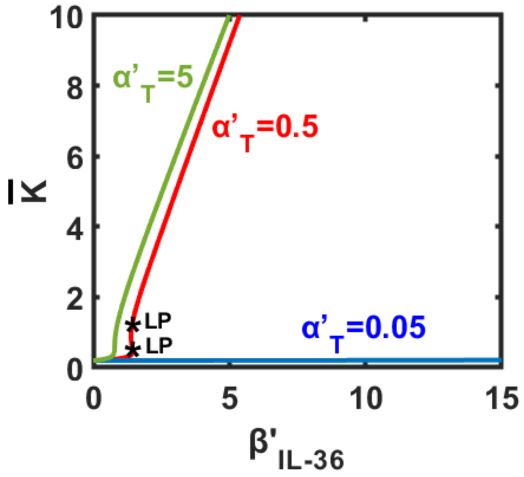

Figure 1: Keratinocytes vs. β'_{IL36}: Comparative graph for varying levels of Effective rates of migration of T- cells towards psoriatic lesion.
